# Engineering bioactive mineralized tumor cells for tumor immunotherapy

**DOI:** 10.3389/fbioe.2025.1582490

**Published:** 2025-04-01

**Authors:** Zikun Shen, Yan He, Ren Mo, Dan Shao

**Affiliations:** ^1^ School of Medicine, South China University of Technology, Guangzhou, Guangdong, China; ^2^ Department of Urology, Inner Mongolia People’s Hospital, Inner Mongolia Urological Institute, Hohhot, Inner Mongolia, China

**Keywords:** whole-cell tumor vaccines, mineralization, bioactive, STING, immunotherapy

## Abstract

**Introduction:**

Whole-cell tumor vaccines are advantageous because of their ability to induce a broad and multifaceted immune response through the presentation of a wide range of tumor antigens, thereby enhancing the ability of the immune system to recognize and target cancerous cells.

**Method:**

In this study, we present a multifunctional vaccine that consists of manganese-mineralized tumor cells and positively charged polymer-immobilized CpG. The Mn^2+^ and CpG released from the engineered vaccine facilitate the maturation of dendritic cells through the activation of the cGAS-STING and TLR9 pathways, respectively.

**Result:**

As a consequence, the engineered vaccine derived from B16F10 cells exhibited a pronounced tumor-suppressive effect, reducing the tumor volume to approximately one-fifth of that in the control group, and significantly extending survival to day 30 in B16F10 tumor-bearing mice. This superior therapeutic outcome is associated with enhanced activation of dendritic cells, increased infiltration of NK and CD8^+^ T cells, and increased production of immune cytokines within the tumor microenvironment.

**Discussion:**

Together, our study highlights the immense potential of engineering bioactive mineralized tumor cells to facilitate whole-cell tumor vaccine-based immunotherapy.

## 1 Introduction

Vaccination represents a cost-effective strategy for cancer prevention and therapy, promoting robust and long-lasting tumor-specific immune responses (Li et al., 2020; Liu et al., 2024). In contrast to nucleic acid or protein subunit vaccines, autologous whole-cell tumor vaccines offer a broader array of tumor-associated antigens, which can elicit potent immune responses against a diverse range of tumor-specific antigens (Diao and Liu, 2023). This characteristic enhances the potential for personalized treatment and obviates the need for neoantigen identification (Pérez-Baños et al., 2023). However, despite their promise, clinical trials of whole-cell vaccines have largely yielded modest outcomes, likely due to challenges related to the loss of immunogenicity and inefficient targeting to antigen-presenting cells (APCs) (Ma et al., 2021). Efforts to address these limitations include physical, chemical, and genetic modifications of tumor cells to render them non-tumorigenic and enhance their immunogenic potential (Gu et al., 2024; Yang et al., 2024). While these strategies show potential, issues related to immunogenicity and the complexity of adjuvant incorporation continue to hinder their broader clinical application. Consequently, there is an urgent need for innovative strategies to engineer whole-cell tumor vaccines that optimize their immunogenicity while minimizing the complexities associated with production.

**SCHEME 1:**
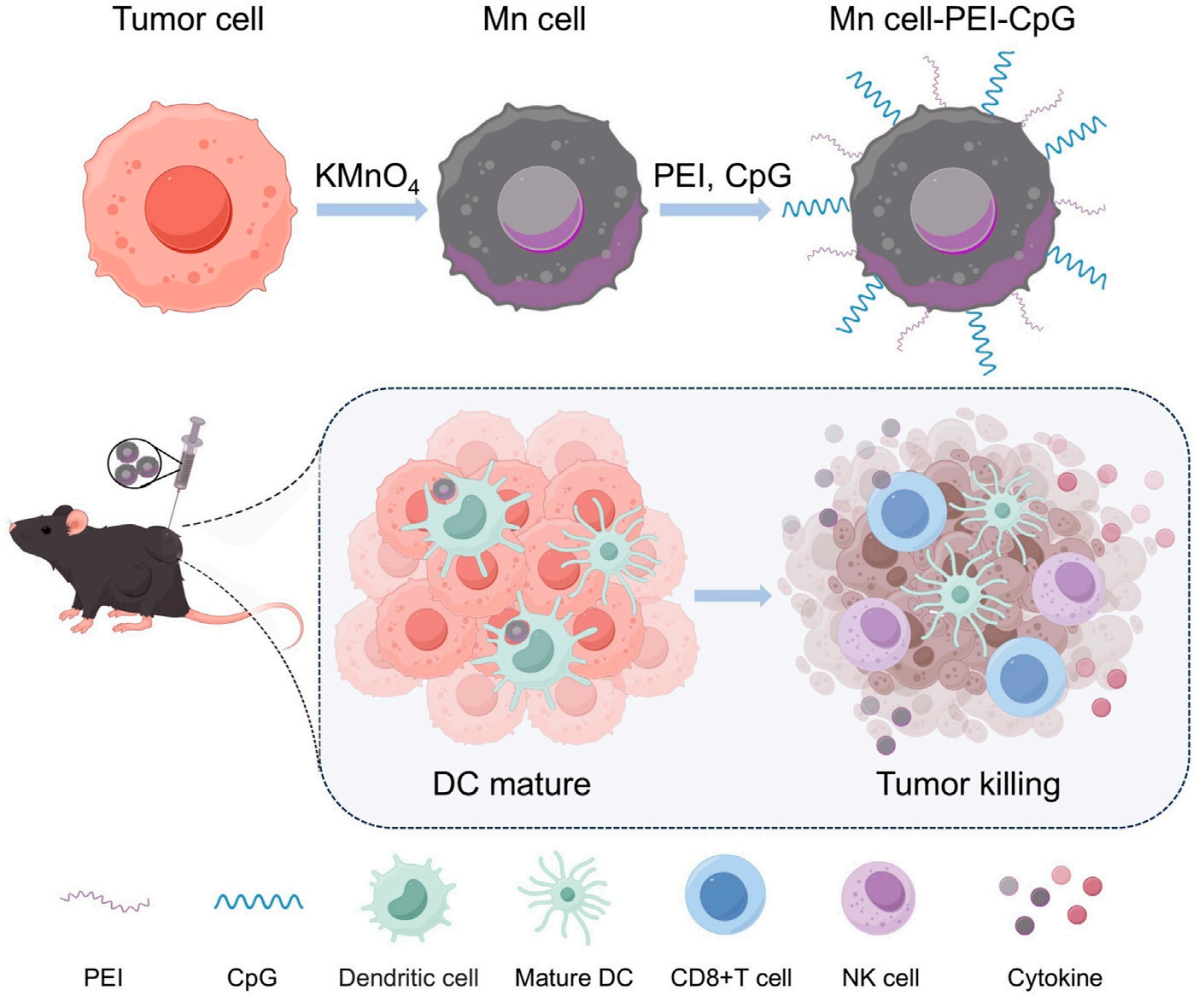
Schematic illustration of the preparation and application of Mn cell-PEI-CpG as a whole-cell tumor vaccine. The scheme was created via Figdraw.

Toll-like receptors (TLRs) expressed on dendritic cells (DCs) play pivotal roles in recognizing pathogen-associated molecular patterns (PAMPs), which are essential for initiating immune responses (Tsan, 2006; Kawai et al., 2024). The incorporation of modified TLR ligands as adjuvants in tumor vaccines significantly enhances immune activation by promoting efficient DC maturation and activation, thereby bridging innate and adaptive immunity (Yang et al., 2022; Xie et al., 2024). These modifications improve antigen presentation, leading to robust T cell activation and the generation of tumor-specific immune responses (Sagiv Barfi et al., 2021). Additionally, TLR ligand modifications can optimize the specificity, intensity, and duration of immune activation, rendering them powerful tools for refining cancer immunotherapies (Chen et al., 2020; Chakraborty et al., 2023). CpG, a well-known TLR9 agonist, enhances antitumor immunity by stimulating DCs to secrete pro-inflammatory cytokines, driving effective T cell responses, and facilitating the recognition of tumor-associated antigens (Sagiv-Barfi et al., 2016). Notably, CpG 2006 has demonstrated promising results as an adjuvant in multiple vaccine candidates (Chagnon et al., 2005; Goldstein et al., 2011; Ngamcherdtrakul et al., 2021), showing encouraging clinical outcomes in human trials (Witzig et al., 2013).

A shell-engineering approach inspired by natural biomineralization processes has been employed to enhance whole-cell tumor vaccines (Guo et al., 2022), improving antigen presentation and promoting dendritic cell activation, leading to stronger and more sustained antitumor immunity (Qin et al., 2024). Manganese dioxide (MnO_2_) enhances tumor immunotherapy by promoting the release of reactive oxygen species (ROS) in the acidic tumor microenvironment, which enhances tumor cell death and activates immune pathways, thereby strengthening immune responses and improving cancer treatment efficacy (Yang et al., 2019; Zhang et al., 2023). Notably, this process enhances the cGAS-STING pathway in immune cells, triggering the production of type I interferons and other pro-inflammatory cytokines that activate both innate and adaptive immune responses (Yang et al., 2021; Gu et al., 2023). The manganese-mineralization of tumor cells offers a novel and effective strategy to enhance whole-cell-based antitumor therapies.

Building on the use of manganese (Mn)-mineralized cells as whole-cell tumor vaccines, we aim to enhance immune activation by incorporating additional ligands for multivalent stimulation. In this study, as shown in Scheme 1, we introduce a method for surface modification of Mn-mineralized tumor cells with the TLR9 agonist CpG, thereby developing an advanced whole-cell tumor vaccine. The MnO_2_-mediated activation of the STING pathway, in combination with CpG-induced activation of the TLR9 pathway, fosters a coordinated immune response that significantly enhances the uptake and activation of APCs. Using a melanoma mouse model, we demonstrate that this vaccination strategy elicits a robust immune response and notably enhances tumor treatment efficacy. Furthermore, we show that Mn-mineralized cells modified with both polyethylenimine (PEI) and CpG adjuvants exhibit superior immune activation and tumor-suppressive effects compared with those of cells with Mn-mineralization alone.

## 2 Results

### 2.1 Preparation and characterization of Mn cell-PEI-CpG

**FIGURE 1:**
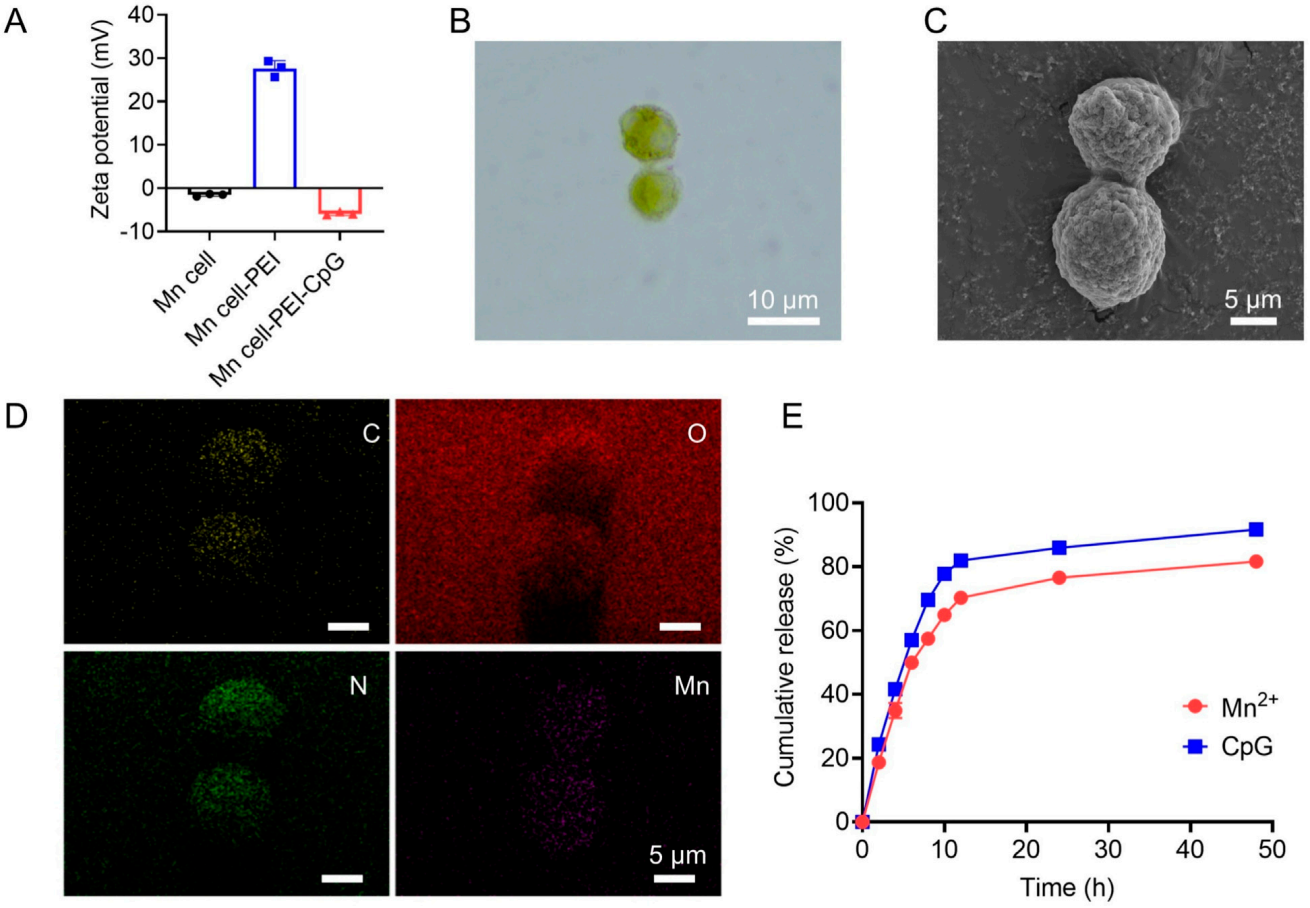
Characterization of Mn cell-PEI-CpG. **(A)** Zeta potential analysis of Mn cell, Mn cell-PEI and Mn cell-PEI-CpG (n = 3). **(B)** Microscope photographs of Mn cell-PEI-CpG. Scale bar, 10 μm. **(C)** SEM images of Mn cell-PEI-CpG. Scale bar, 5 μm. **(D)** EDS analysis of Mn cell-PEI-CpG. Scale bar, 5 μm. **(E)** CpG and Mn^2+^ release profiles from the Mn cell-PEI-CpG vaccine in PBS (pH 5.5) (n = 3). The data are presented as the means ± s.d.

To prepare Mn-mineralized cells (Mn cell), tumor cells were suspended in a potassium permanganate (KMnO_4_) solution and incubated at room temperature for 15 min. KMnO_4_ undergoes redox reactions with reducing substances within cells, such as glucose and glycerophospholipids, and interacts with negatively charged groups on the cell surface, including phosphate, carboxyl, and sulfate groups, resulting in the formation of stable complexes or mineralized layers. Under physiological conditions, both the Mn-mineralized cells and CpG adjuvants are negatively charged. To increase the efficacy of adjuvant modification, PEI was introduced to adsorb onto the surface of the Mn-mineralized cells, imparting a positive charge. This facilitated the uniform binding of CpG to the cell surface. PEI, a cationic organic polymer, has been reported as a ligand for TLR4 and TLR5 and is known to enhance the immunogenicity of tumor vaccines (Ma and Yang, 2010; Kuai et al., 2018). As shown in Figure 1A, the experimental results of zeta potential analysis confirmed that the Mn-mineralized cells were effectively and uniformly coated with PEI and CpG in succession, forming Mn-mineralized cells carrying PEI and CpG adjuvants (Mn cell-PEI-CpG). The Mn cell-PEI-CpG exhibited a brownish-yellow color under optical microscopy, confirming the successful and uniform attachment of manganese to the surface of B16F10 cells following treatment with KMnO_4_ (Figure 1B). Scanning electron microscopy (SEM) revealed that the morphology of the treated cells was intact (Figure 1C). As shown in Figure 1D, the uniform adsorption of Mn on the cell surface was further confirmed by energy dispersive X-ray spectroscopy (EDS). The release profiles of CpG and Mn^2+^ from the Mn cell-PEI-CpG vaccine were then evaluated under simulated endosomal pH conditions (pH 5.5). As shown in Figure 1E, CpG and Mn^2+^ exhibited comparable release profiles at pH 5.5, indicating coordinated release behavior.

**FIGURE 2:**
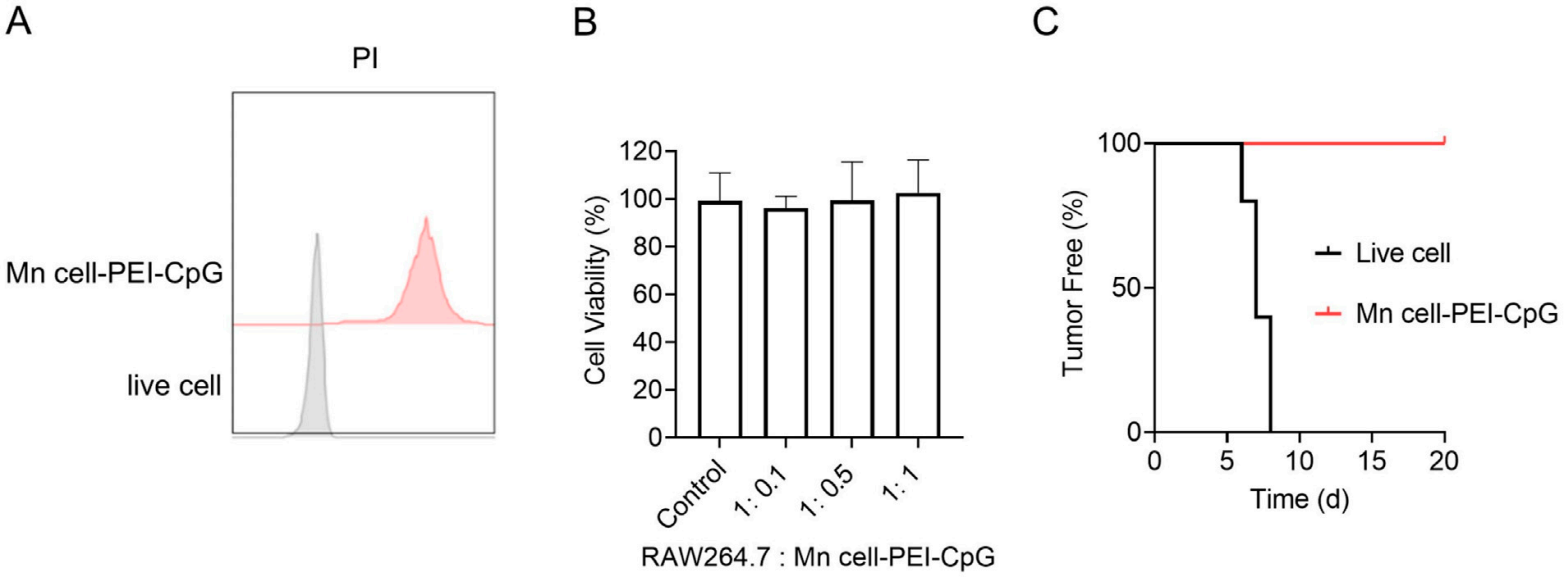
Characterization of the safety of Mn cell-PEI-CpG. **(A)** Representative mean fluorescence intensity (MFI) histogram of live cells and Mn cell-PEI-CpG with propidium iodide (PI). **(B)** Cytotoxicity of Mn cell-PEI-CpG (n = 5). **(C)** Tumor-free curve of C57BL/6 mice inoculated on day 0 with either live or engineered B16F10 cells (n = 5). The tumor-free data represent the percentage of mice that remained tumor-free. The data are presented as the means ± s.d.

The proliferative capacity and tumorigenicity of tumor cells remain critical concerns in the development of whole-cell tumor vaccines. To assess the potential oncogenic risks and *in vitro* safety of Mn cell-PEI-CpG, we first evaluated cell viability via propidium iodide (PI) staining. PI, a nuclear dye, permeates only compromised cell membranes and binds to DNA, thereby indicating cell death. The PI staining results revealed a 100% PI-positive rate in Mn cell-PEI-CpG, with no live-cell fluorescence detected, whereas live cells exhibited no PI staining (Figure 2A). To further assess the potential cytotoxicity of Mn cell-PEI-CpG, Raw264.7 macrophages were incubated with varying concentrations of Mn cell-PEI-CpG for 24 h, and cell viability was measured. The results revealed no cytotoxic effects on Raw264.7 macrophages at Mn cell-PEI-CpG ratios ranging from 1:0.1 to 1:1 (Figure 2B). To investigate the safety of Mn cell-PEI-CpG *in vivo*, healthy female C57BL/6 mice were inoculated with live B16F10 cells and the Mn cell-PEI-CpG vaccine in the left axillary region, and tumor formation was monitored over time. Tumor growth was evident in the live cell group between days 5 and 8, whereas no subcutaneous tumor nodules were observed in the Mn cell-PEI-CpG vaccine group until day 20 (Figure 2C). These results suggest that Mn cell-PEI-CpG does not exhibit significant tumorigenic potential *in vivo*, supporting its safety for use as a therapeutic vaccine.

### 2.2 Mn cell-PEI-CpG enhances phagocytosis and immune activation of dendritic cells

**FIGURE 3:**
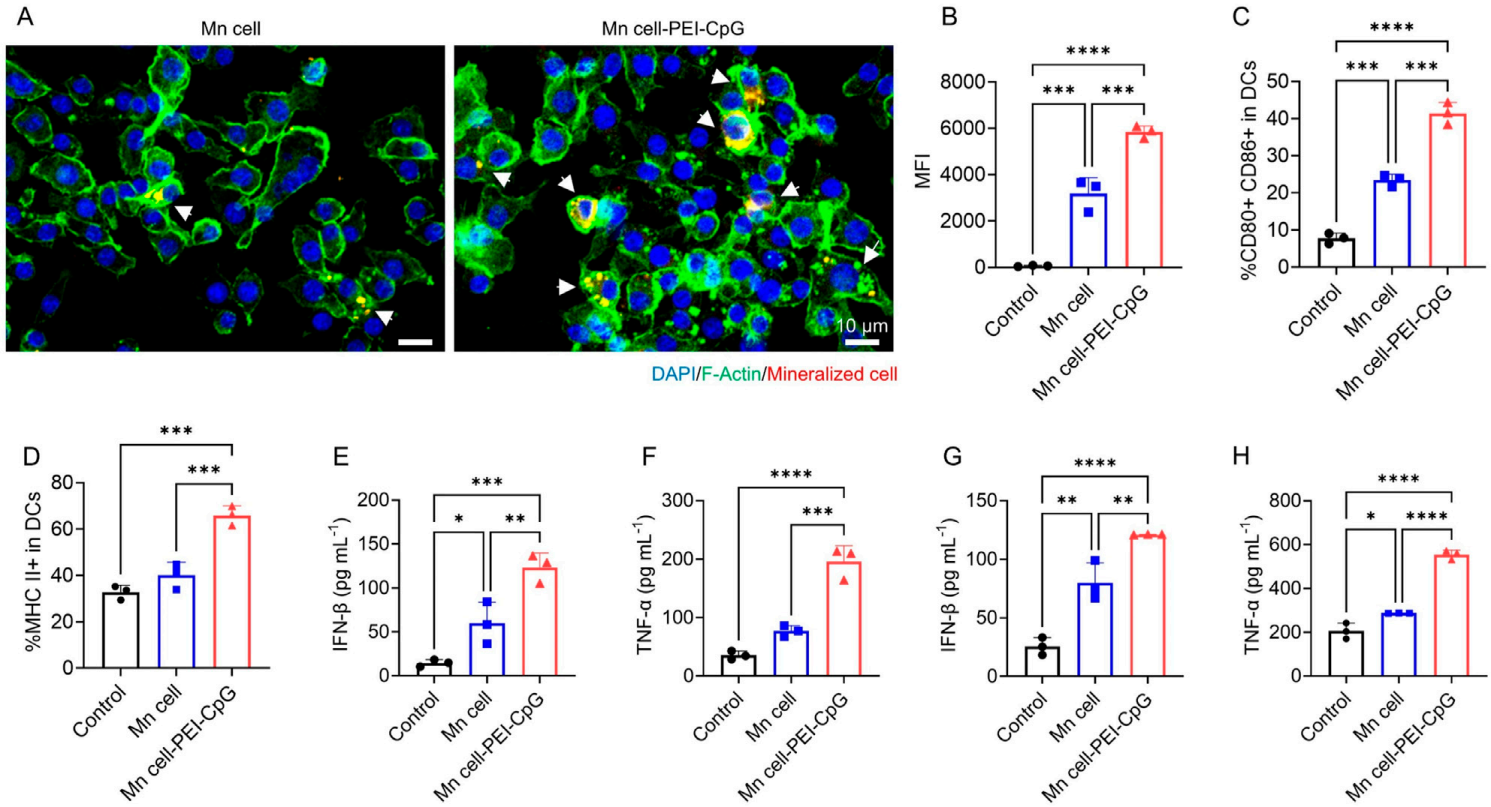
*In vitro* APC uptake and activation of engineered tumor cells. **(A)** Representative fluorescence images of BMDCs incubated with DiD-labeled engineered tumor cells. Scale bar, 10 μm. **(B)** Flow cytometry analysis of BMDC uptake by engineered tumor cells (n = 3). **(C)** Flow cytometry analysis of the activation of BMDCs after different treatments using CD80 and CD86 as DC maturation markers (n = 3). **(D)** Flow cytometry analysis of MHC II-positive BMDCs (n = 3). **(E)** Determination of IFN-β secretion in the supernatant of BMDCs (n = 3). **(F)** Determination of TNF-α secretion in the supernatant of BMDCs (n = 3). **(G)** Determination of IFN-β secretion in the supernatant of BMDMs (n = 3). **(H)** Determination of TNF-α secretion in the supernatant of BMDMs (n = 3). The data are presented as the means ± s.d. Statistical significance was calculated via one-way ANOVA with Tukey’s *post hoc* test. *P < 0.05, **P < 0.01, ***P < 0.001, ****P < 0.0001.

To activate both innate immunity and tumor-specific adaptive immune responses, tumor vaccines must be efficiently internalized, processed, and presented by APCs in tumors. Consequently, the capacity of a vaccine to be taken up by APCs and stimulate their maturation is a critical factor for its effectiveness. We first assessed the internalization of Mn cell-PEI-CpG and Mn cell by bone marrow-derived dendritic cells (BMDCs) using confocal microscopy and flow cytometry. Tumor cells were labeled with the DiD membrane probe prior to manganese mineralization, enabling visualization of interactions between the DiD fluorescence signal and the cytoskeleton via confocal microscopy to assess the uptake of manganese-mineralized cells by macrophages. Quantitative analysis of cellular uptake was further conducted via flow cytometry by measuring the average fluorescence intensity of the DiD signal. After co-culture with BMDCs for 4 h, the vaccines were internalized by BMDCs, with Mn cell-PEI-CpG demonstrating significantly enhanced phagocytosis relative to that of Mn cell (Figure 3A). Notably, BMDC uptake of Mn cell-PEI-CpG was 1.7 times higher than that of Mn cell, highlighting the substantial impact of PEI and CpG modifications on improving vaccine uptake efficiency (Figure 3B).

We further investigated the activation and maturation of BMDCs by analyzing the expression of surface costimulatory molecules (CD80 and CD86) and major histocompatibility complex (MHC) class II molecules through flow cytometry. Our results revealed that Mn cell-PEI-CpG-treated BMDCs presented a significantly increased population of mature cells (CD80^+^ CD86^+^) and increased expression of MHC class II molecules (Figures 3C, D). Additionally, Mn cell-PEI-CpG treatment led to the highest levels of pro-inflammatory cytokine secretion, including interferon-beta (IFN-β) and tumor necrosis factor-alpha (TNF-α) (Figures 3E, F). Notably, compared with manganese-mineralized cells without adjuvant modification, adjuvant-modified manganese-mineralized cells produced 2.06 times more IFN-β and 2.54 times more TNF-α. Furthermore, we investigated the impact on macrophage activation and found that macrophages co-incubated with Mn cell-PEI-CpG presented the highest levels of IFN-β and TNF-α secretion (Figures 3G, H). These findings suggest that Mn cell-PEI-CpG acts as a potent immune-stimulating agent by activating APCs, promoting antigen presentation, and driving pro-inflammatory cytokine secretion.

### 2.3 Efficacy of the Mn cell-PEI-CpG vaccine in inhibiting tumor progression

**FIGURE 4:**
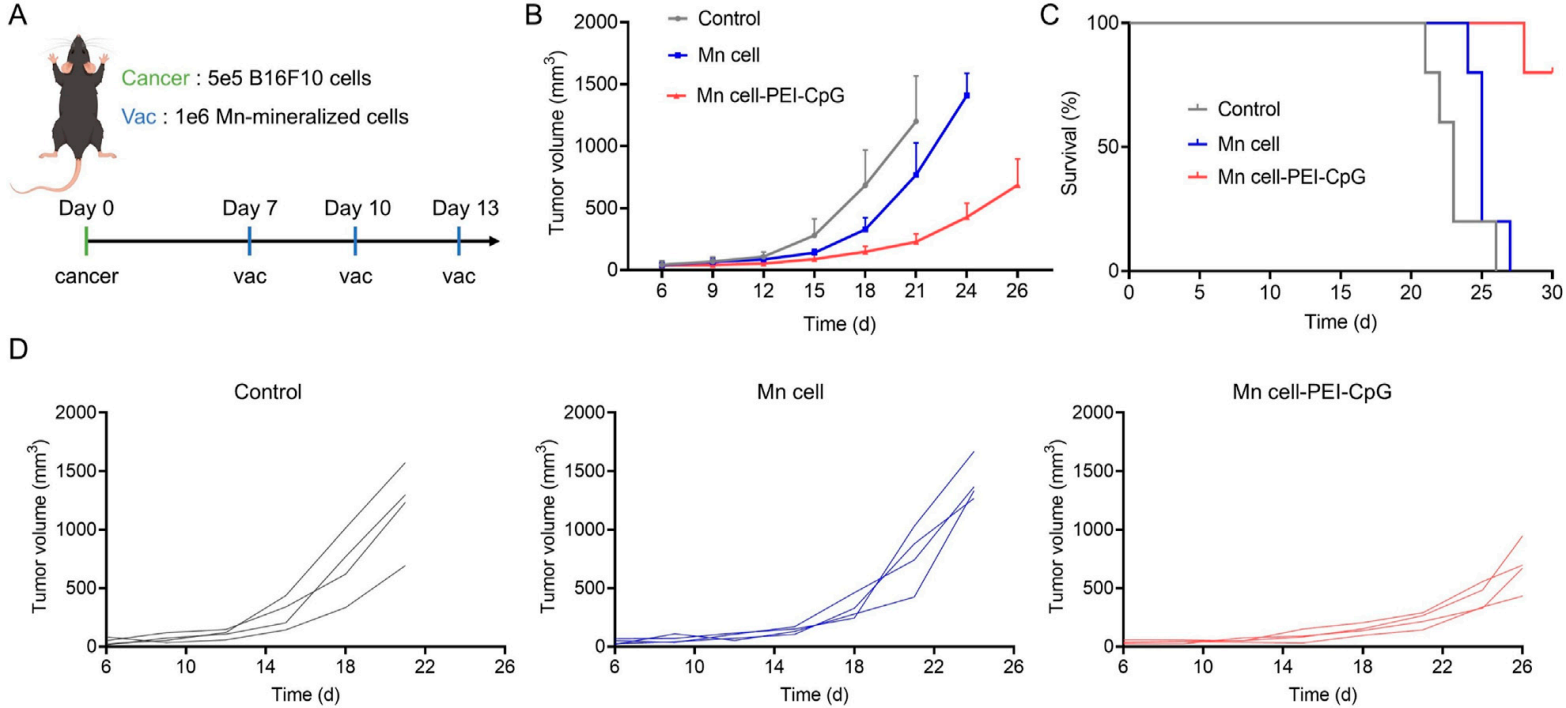
Therapeutic efficacy of engineered tumor cells as tumor vaccines. **(A)** Schedule for the therapeutic efficacy study. Following B16F10 tumor inoculation on day 0, the engineered cells were intratumorally injected on days 7, 10, and 13. **(B)** The average tumor growth curves of B16F10 tumors after various treatments (n = 4). **(C)** Survival outcomes of mice post-treatment (n = 4). **(D)** Individual tumor growth curves of each group receiving various treatments. The data are presented as the means ± s.d.

Building on the promising results suggesting that Mn cell-PEI-CpG functions as an immune-stimulating agent, we evaluated its therapeutic efficacy as a tumor vaccine in a B16F10 melanoma model. After the subcutaneous inoculation of B16F10 cells on day 0, mice were administered intratumoral injections of Mn-mineralized cells on days 7, 10, and 13, with consistent doses of Mn-mineralized cells (Figure 4A). As shown in Figure 4B, immunization with Mn cell resulted in a moderate inhibitory effect on tumor growth compared with that in the control group. In contrast, Mn cell-PEI-CpG significantly reduced B16F10 tumor growth. Survival analysis revealed that immunization with Mn cell-PEI-CpG prolonged the survival time to 30 days, significantly outperforming the other groups (Figure 4C). Notably, three out of four mice in the Mn cell-PEI-CpG group survived until day 30. Individual tumor growth curves further demonstrated that tumor progression in the Mn cell-PEI-CpG-treated group was significantly suppressed (Figure 4D).

### 2.4 Treatment with Mn cell-PEI-CpG induced a robust immune response

**FIGURE 5:**
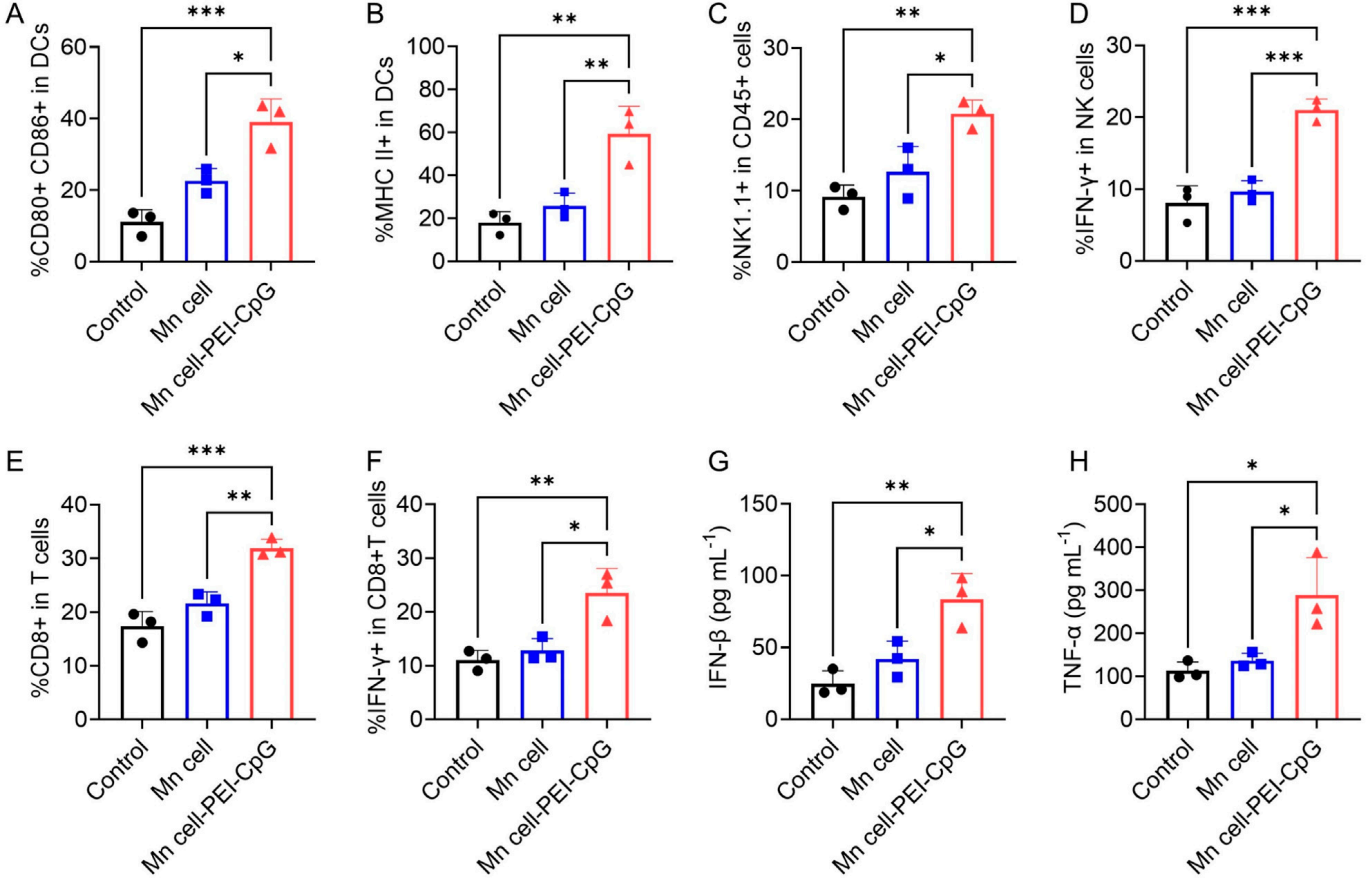
Immune responses activated by engineered whole-cell tumor vaccines in established B16F10 tumor models. **(A)** Flow cytometry analysis of mature DCs in the TME after different treatments (n = 3). **(B)** Flow cytometry analysis of MHC II-positive DCs in the TME (n = 3). **(C)** Flow cytometry analysis of NK cells in the TME after different treatments (n = 3). **(D)** Flow cytometry analysis of IFN-γ-positive NK cells in the TME (n = 3). **(E)** Flow cytometry analysis of CD8^+^ T cells in the TME of mice receiving different treatments (n = 3). **(F)** Flow cytometry analysis of IFN-γ-positive CD8^+^ T cells in the TME (n = 3). **(G)** Determination of IFN-β secretion in the TME (n = 3). **(H)** Determination of TNF-α secretion in the TME (n = 3). The data are presented as the means ± s.d. Statistical significance was calculated via one-way ANOVA with Tukey’s *post hoc* test. *P < 0.05, **P < 0.01, ***P < 0.001, ****P < 0.0001.

The impact of Mn cell-PEI-CpG on the tumor microenvironment (TME) was assessed by flow cytometric analysis of tumor-infiltrating lymphocytes on day 16. DCs play a pivotal role in the TME by capturing, processing, and presenting tumor antigens, thereby initiating T cell activation and antitumor immune responses (Wculek et al., 2020; Moon et al., 2025). Following three intratumoral injections of Mn cell-PEI-CpG, a significant increase in mature DCs (CD80^+^ CD86^+^) and DCs expressing MHC class II molecules was observed, indicating enhanced antigen presentation and the activation and maturation of APCs within the TME (Figures 5A, B). Tumor-infiltrating natural killing (NK) cells, along with the IFN-γ they secrete, play a critical role in antitumor immunity (Cózar et al., 2021). NK cells directly recognize and kill tumor cells, while IFN-γ further enhances the activity of APCs, promotes T cell-mediated antitumor responses, and upregulates MHC molecule expression on tumor cells, improving immune system recognition of tumors (Wu et al., 2020). Moreover, IFN-γ can inhibit tumor angiogenesis and reshape the TME to favor immune cell infiltration and function, thus synergistically enhancing the antitumor immune response (Gosselin et al., 1999). Compared with those in the control and Mn cell-treated groups, tumors treated with Mn cell-PEI-CpG presented a significantly greater proportion of NK cells and IFN-γ-secreting NK cells, indicating enhanced NK cell infiltration and strengthened nonspecific immunity in the TME (Figures 5C, D). Tumor-infiltrating CD8^+^ T cells, along with the IFN-γ they produce, are essential for direct tumor cell killing and the enhancement of antitumor immunity (Lin et al., 2025). As key effector cells in antitumor immunotherapy, the infiltration of CD8^+^ T cells and their secretion of IFN-γ were significantly elevated in Mn cell-PEI-CpG-treated tumors, further supporting the promotion of a robust antitumor immune response (Figures 5E, F). Simultaneously, elevated levels of the pro-inflammatory cytokines IFN-β and TNF-α were detected in the Mn cell-PEI-CpG treatment group (Figures 5G, H). In summary, Mn cell-PEI-CpG treatment led to the activation of multiple immune cell populations and a strong antitumor immune response, resulting in effective tumor suppression.

**FIGURE 6:**
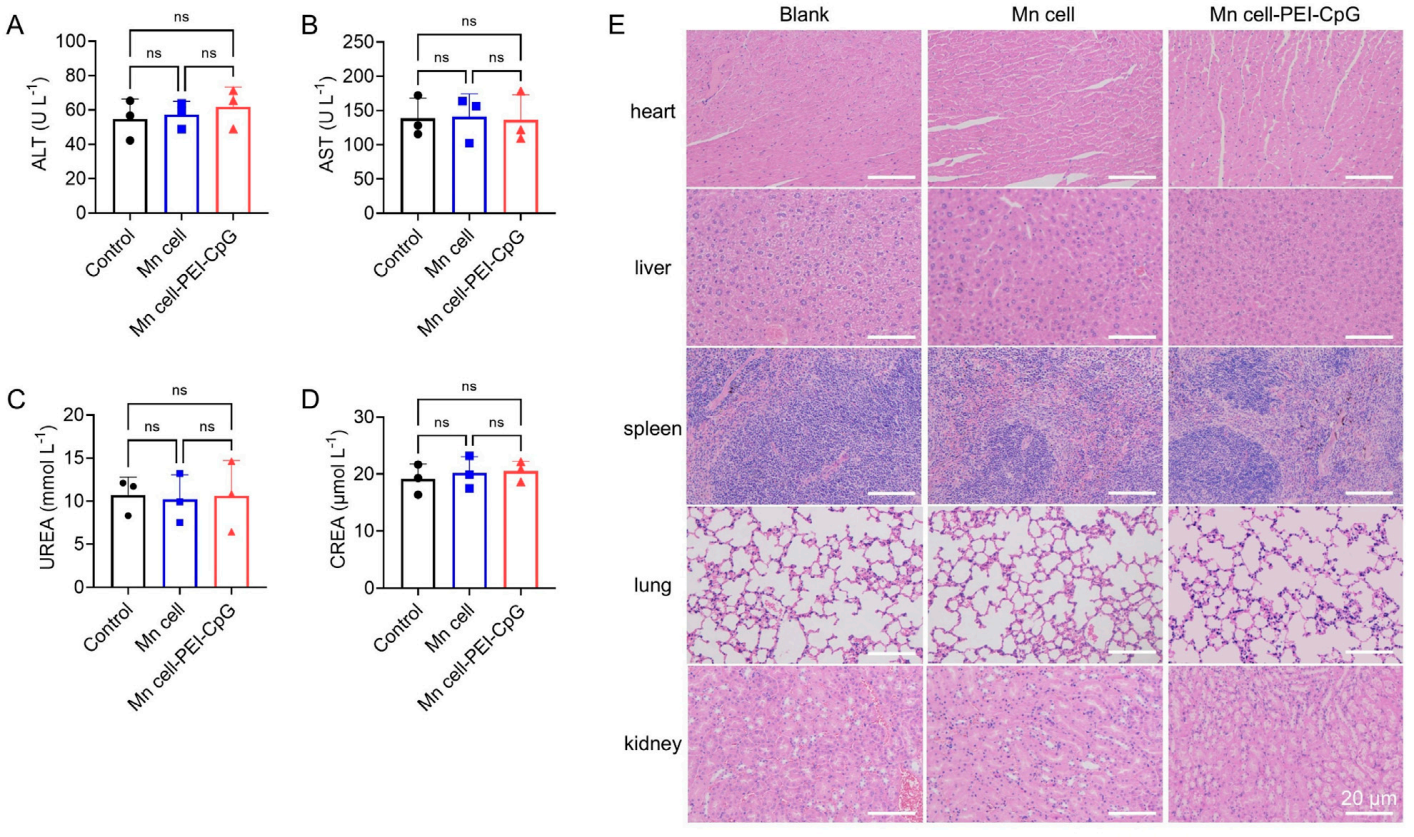
Toxicology studies of engineered whole-cell tumor vaccines. The serum levels of **(A)** aspartate transaminase (ALT), **(B)** alanine aminotransferase (AST), **(C)** urea nitrogen (UREA) and **(D)** serum creatinine (CREA) after various treatments (n = 3). **(E)** Representative images of H&E-stained major organ sections after various treatments. Scale bar, 20 μm. The data are presented as the means ± s.d. Statistical significance was calculated via one-way ANOVA with Tukey’s *post hoc* test. ns, not significant.

Finally, toxicology studies of engineered whole-cell tumor vaccines were conducted. The mice were euthanized on day 30 for serum biochemistry analysis and histological assessment. No significant hepatic or renal toxicity was observed, as evidenced by normal levels of liver function markers and kidney function markers (Figures 6A–D). Hematoxylin and eosin (H&E) staining revealed no apparent lesions or abnormalities in any of the examined organs, indicating the absence of significant systemic toxicity following treatment with Mn cell and Mn cell-PEI-CpG (Figure 6E).

## 3 Discussion

In this study, we engineered PEI and CpG as adjuvants on the surface of MnO_2_-mineralized tumor cells to leverage the synergistic activation of the cGAS-STING and multivalent TLR pathways for immune modulation. Compared to cells with pure manganese mineralization, Mn cell-PEI-CpG exhibited superior uptake and activation of APCs and facilitated the presentation of tumor cell-derived antigenic peptides. It also induced more than double the secretion of pro-inflammatory cytokines TNF-α and IFN-β. The elevated secretion of IFN-β is likely linked to the MnO_2_-mediated cGAS-STING signaling pathway on the surface of the tumor vaccine. We hypothesize that the enhanced uptake efficiency of Mn cell-PEI-CpG-treated cells contributes to the greater secretion of IFN-β than that of Mn cell-treated cells. Intratumoral injection of Mn cell-PEI-CpG resulted in significant inhibition of B16F10 tumor growth and increased mouse survival rates. This therapeutic effect was associated with increased infiltration of DCs, NK cells, and CD8^+^ T cells, as well as a marked elevation in immune cytokines within the TME. These findings suggest that Mn cell-PEI-CpG, as an immunogenicity-enhanced whole-cell tumor vaccine, not only activates APCs but also promotes immune cell infiltration into the TME without introducing tumorigenic risk or biological toxicity. This underscores its potential as a promising strategy for the development of tumor vaccines.

## 4 Materials and methods

### 4.1 Materials

CpG oligonucleotide 2006 was purchased from InvivoGen, and PEI was purchased from Sigma‒Aldrich. The PI staining solution, DAPI staining solution, Actin-Tracker Green-488 and Cell Plasma Membrane Staining Kit with DiD were obtained from Beyotime Biotechnology.

TNF-α ELISA Kit and IFN-β ELISA Kit were purchased from Biolegend. Antibodies, including TruStain FcX™ (anti-mouse CD16/32), APC/Cy7-CD45, PerCP/Cy5.5-CD11c, APC-CD86, PE/D549-CD80, Alexa Fluor 700-MHC Ⅱ, FITC-NK1.1, BV421-CD3, PE/Cy7-CD4, BV711-CD8, and PE-IFN-γ, were purchased from Biolegend. A fixation/permeabilization kit was obtained from BD Bioscience.

### 4.2 Preparation of Mn cell-PEI-CpG

Approximately 1 × 10^6^ viable B16F10 cells were suspended in 5 mL of PBS and incubated with a 0.2 mg mL^-1^ KMnO_4_ solution at room temperature to facilitate the formation of a manganese mineralization layer. After 15 min of mineralization, the manganese-treated cells were rinsed twice with PBS and resuspended in 1 mL of a 0.2 mg mL^-1^ PEI solution. Following a 10-min incubation with gentle agitation, the cells were washed twice with PBS. For CpG 2006 adsorption, the cells were incubated with 2 mg mL-1 CpG 2006 in PBS, using a similar protocol.

### 4.3 Characterization of Mn cell-PEI-CpG

The engineered cells were fixed in 2.5% glutaraldehyde in 0.1 M phosphate buffer and incubated overnight at 4 °C. Following fixation, the cells underwent a graded ethanol dehydration protocol (30%, 50%, 70%, 90%, 95%, and 100%) with 15-min incubations at each concentration, culminating in a final immersion in 100% ethanol for complete dehydration. After a subsequent overnight drying period, the cells were sputter-coated with a platinum conductive layer and examined using scanning electron microscopy (SEM, Merlin, Zeiss).

### 4.4 Evaluation of cellular uptake

To visualize the interaction between BMDCs and engineered cells, B16F10 cells were labeled with DiD for 30 min. The BMDCs were cultured on a glass-bottom plate for 24 h, after which the engineered cells were added and co-incubated with the BMDCs for 4 h. After incubation, the cells were fixed in 4% paraformaldehyde in PBS for 10 min. After fixation, the cells were permeabilized with 0.1% Triton-X in PBS for 15 min and blocked with 1% BSA for 20 min. The cytoskeleton was stained with Actin-Tracker Green-488, and nuclei were counterstained with DAPI. Imaging was conducted using a confocal fluorescence microscope (LSM 880 with Airyscan, ZEISS), and image processing was performed using ZEN Lite software (v.3.10).

For quantification of cellular uptake, BMDCs were cultured for 24 h, followed by co-incubation with engineered cells labeled with DiD for 4 h. The cultures were then washed with PBS to remove any unbound engineered cells. The remaining BMDCs were collected, and the fluorescence of the mineralized cells was analyzed via flow cytometry (LSRFortessa, BD).

### 4.5 *In vitro* BMDC maturation assay and cytokine profiling

BMDCs were exposed to Mn-cell or Mn cell-PEI-CpG for 24 h. After incubation, the cells were harvested for flow cytometric analysis, and the supernatants were collected for cytokine profiling. For flow cytometry, BMDCs were first blocked with anti-CD16/32 antibodies and then stained with fluorophore-conjugated antibodies against CD11c, CD86, CD80, and MHC II for 30 min. Flow cytometry analysis was performed to assess cell maturation. The concentrations of the cytokines TNF-α and IFN-β in the supernatants were measured using ELISA kits.

### 4.6 Therapeutic vaccine administration

C57BL/6 mice (6 weeks old) were subcutaneously inoculated with 5 × 10^5^ B16F10 cells. Tumor-bearing mice were randomly assigned to one of three groups: (I) control, (II) Mn cell, or (III) Mn cell-PEI-CpG. An intratumoral injection of the vaccines was administered on days 7, 10, and 13. The tumor volume was calculated using the formula: volume = length × width^2^ × 0.5. Tumor size was monitored every 3 days, and the mice were euthanized when the tumor volume reached 1.5 cm^3^ or when they exhibited significant weight loss or unhealing ulceration. All animals received humane care following the Guide for the Care and Use of Laboratory Animals, and the study was approved by the Animal Care and Use Committee of South China University of Technology (ACE2019031).

### 4.7 Tumor immune analysis

For immune cell analysis within the tumors, tumor tissues were collected on day 16 and homogenized in cold PBS containing digestive enzymes to generate single-cell suspensions. The cell suspensions were incubated with anti-CD16/32 antibodies to block Fc receptors, followed by staining with fluorophore-conjugated antibodies against CD45, CD11c, CD80, CD86, MHC II, CD3, CD4, CD8, and NK1.1 for 30 min at 4°C. After staining, the cells were fixed and permeabilized with appropriate solutions for detection of the intracellular marker IFN-γ. Flow cytometry was subsequently performed to analyze the immune cell profiles.

### 4.8 Statistical analysis

The data are presented as the means ± s.d. Comparisons across multiple groups were conducted via two-tailed one-way analysis of variance (ANOVA) followed by Tukey’s *post hoc* tests via GraphPad Prism software (version 9.5.1.733). Statistical significance was defined as P < 0.05.

## Data Availability

The raw data supporting the conclusions of this article will be made available by the authors, without undue reservation.
