# Supplementary material for: Engineering bioactive mineralized tumor cells for tumor immunotherapy
Source: Front Bioeng Biotechnol. 2025 Apr 1;13:1582490. doi: 10.3389/fbioe.2025.1582490 (PMC12023274; doi:10.3389/fbioe.2025.1582490)
Supplement: Supplementary file 1 [file DataSheet1.docx]

Supplementary Material

Engineering Bioactive Mineralized Tumor Cells for Tumor Immunotherapy

Zikun Shen^1^, Yan He^1^, Ren Mo^2*^, Dan Shao^1*^

^1^School of Medicine, South China University of Technology, Guangzhou, Guangdong, 510006, China

^2^Department of Urology, Inner Mongolia People’s Hospital, Inner Mongolia Urological Institute, Hohhot, Inner Mongolia, 010017, China

***Correspondence:**Prof. Ren Mo, Department of Urology, Inner Mongolia People’s Hospital, Inner Mongolia Urological Institute, Hohhot, Inner Mongolia, 010017, China. E-mail: moren325@163.com.

Prof. Dan Shao, School of Medicine, South China University of Technology, Guangzhou, Guangdong, 510006, China. E-mail: shaodan@scut.edu.cn

# Experimental methods

## Viability of Mn Cell-PEI-CpG

The engineered cells were incubated with a 0.5 μmol L⁻¹ propidium iodide (PI) staining solution for 15 minutes at room temperature. Following this, the supernatant was discarded, and the cells were washed three times with PBS to remove any unbound dye. Flow cytometry was then performed to assess the extent of PI staining.

## Cytotoxicity studies of Mn Cell-PEI-CpG

Cytotoxicity was evaluated using the sulforhodamine B (SRB) assay. RAW264.7 cells were co-incubated with Mn Cell-PEI-CpG for 24 hours, followed by cell fixation and staining with SRB dye. The absorbance of the dye, which binds to cellular proteins, was measured to quantify cell viability. This approach provides an indirect assessment of Mn Cell-PEI-CpG-induced cytotoxicity by correlating protein content with cell survival.

## Isolation and Culture of Bone Marrow-Derived Dendritic Cells (BMDCs)

BMDCs were isolated from C57BL/6 mice and cultured in RPMI-1640 complete medium supplemented with GM-CSF at 20 ng mL^-1^ and IL-4 at 5 ng mL^-1^ for 7 days.

# Supplementary Figures


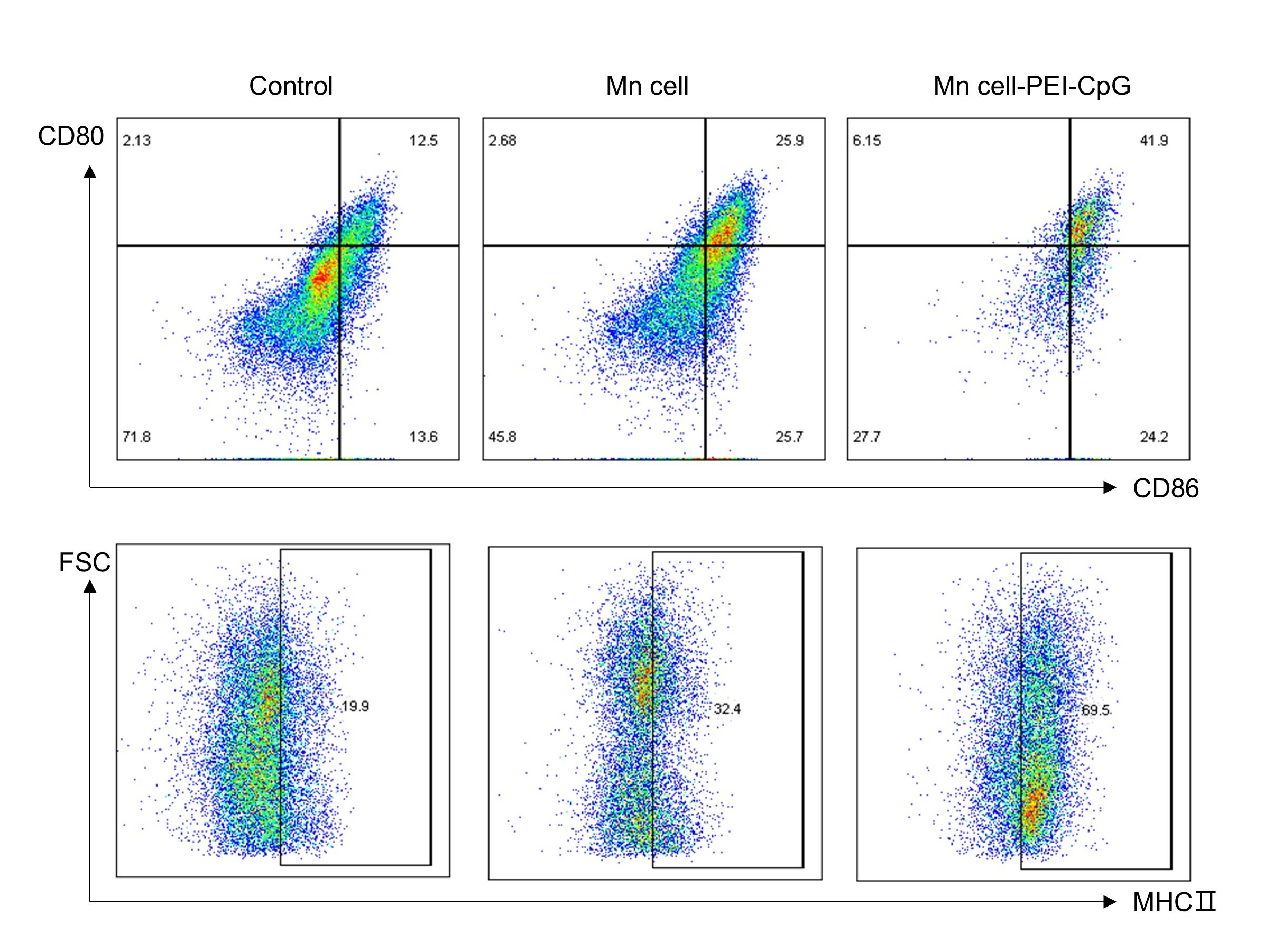


**Supplementary Figure 1.** Representative flow cytometry analysis images of mature DCs (CD80+ CD86+) and MHC Ⅱ-positive DCs in TME (gated on CD11c+ cells).


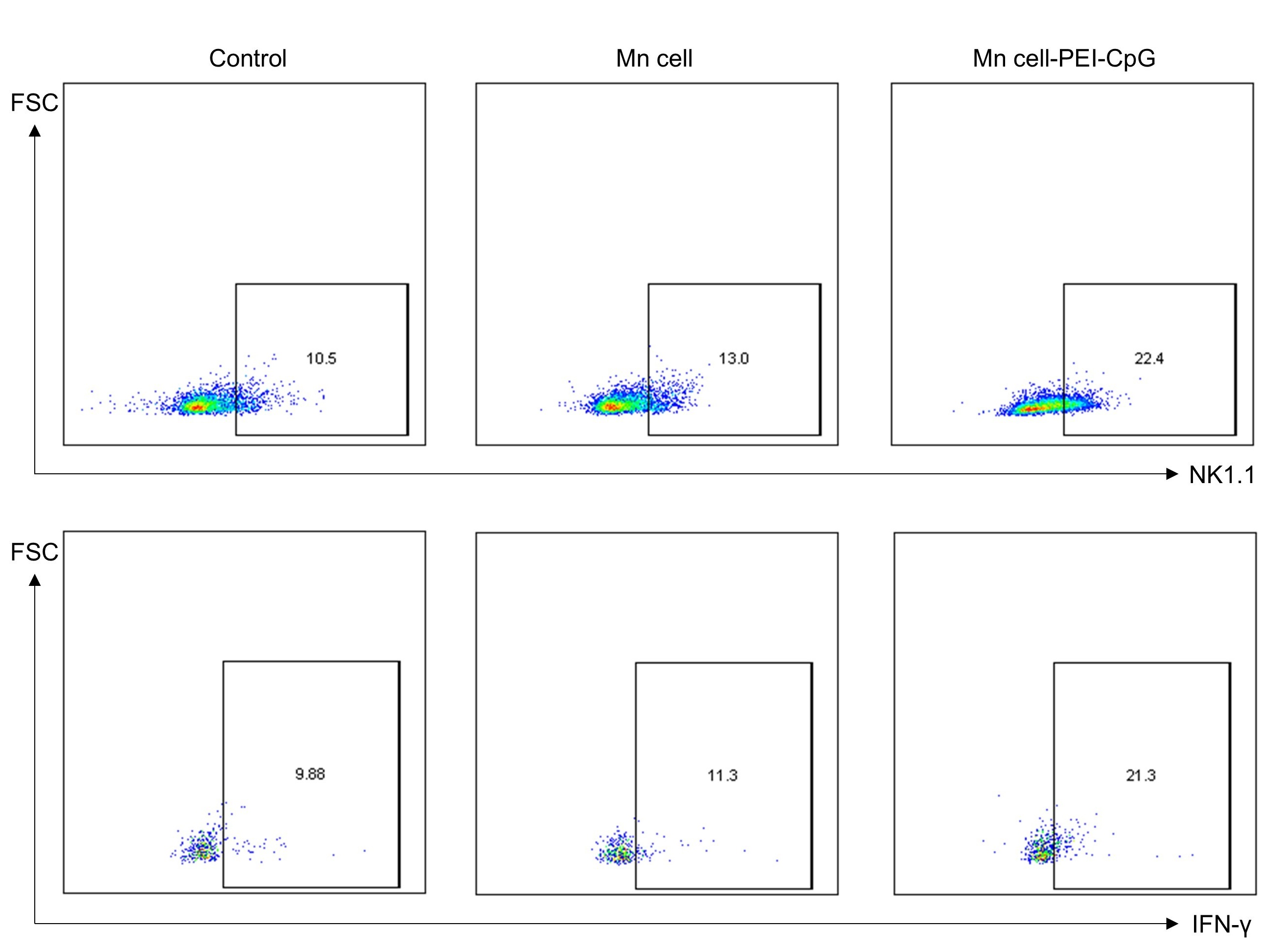


**Supplementary Figure 2.** Representative flow cytometry analysis images of NK cells and IFN-gamma-positive NK cells (gated on NK1.1+ cells) in TME.


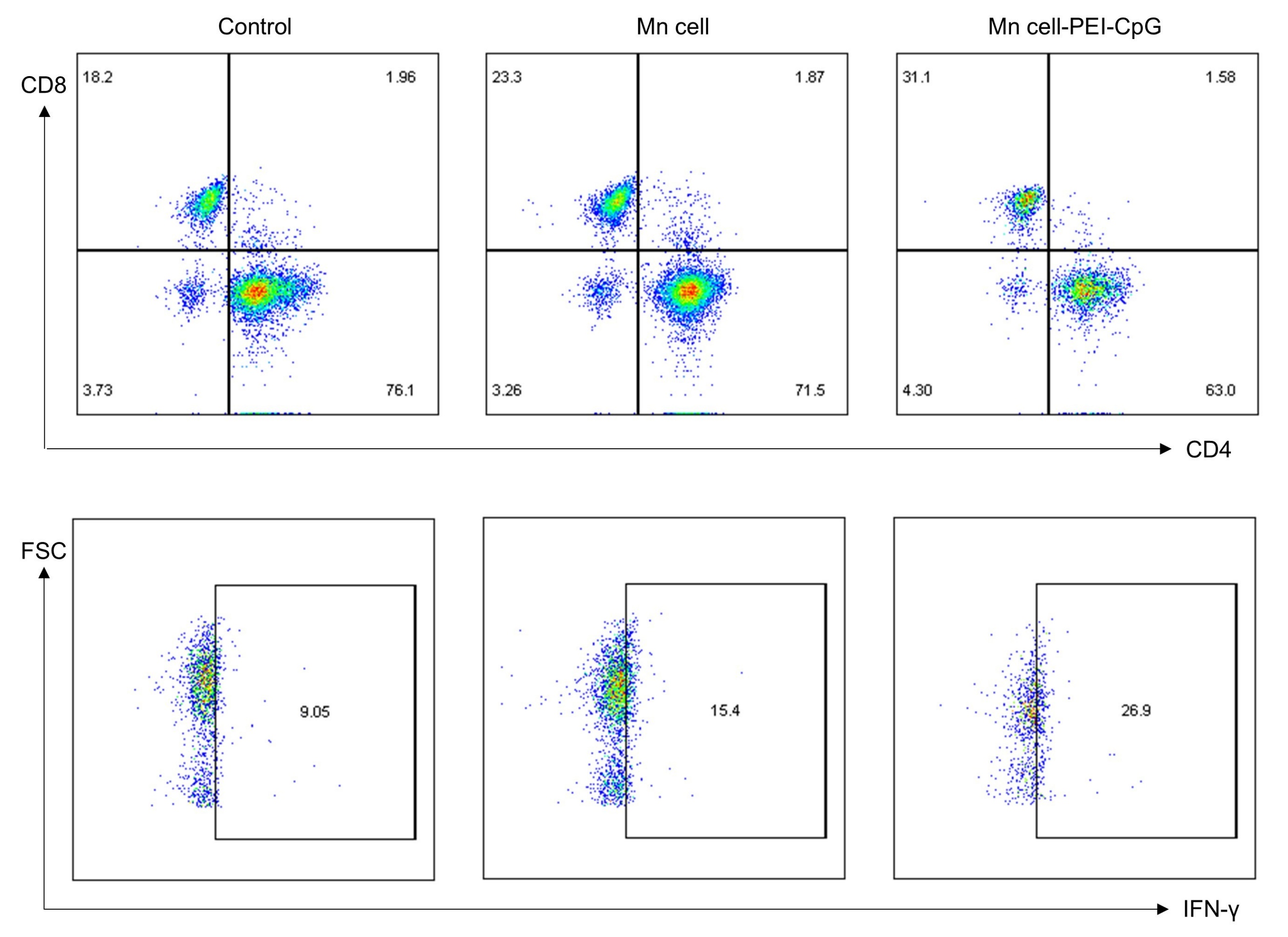


**Supplementary Figure 3.** Representative flow cytometry analysis images of CD8+ T cells (gated on CD3+ T cells) and IFN-gamma-positive CD8+ T cells (gated on CD8+ T cells) in TME.


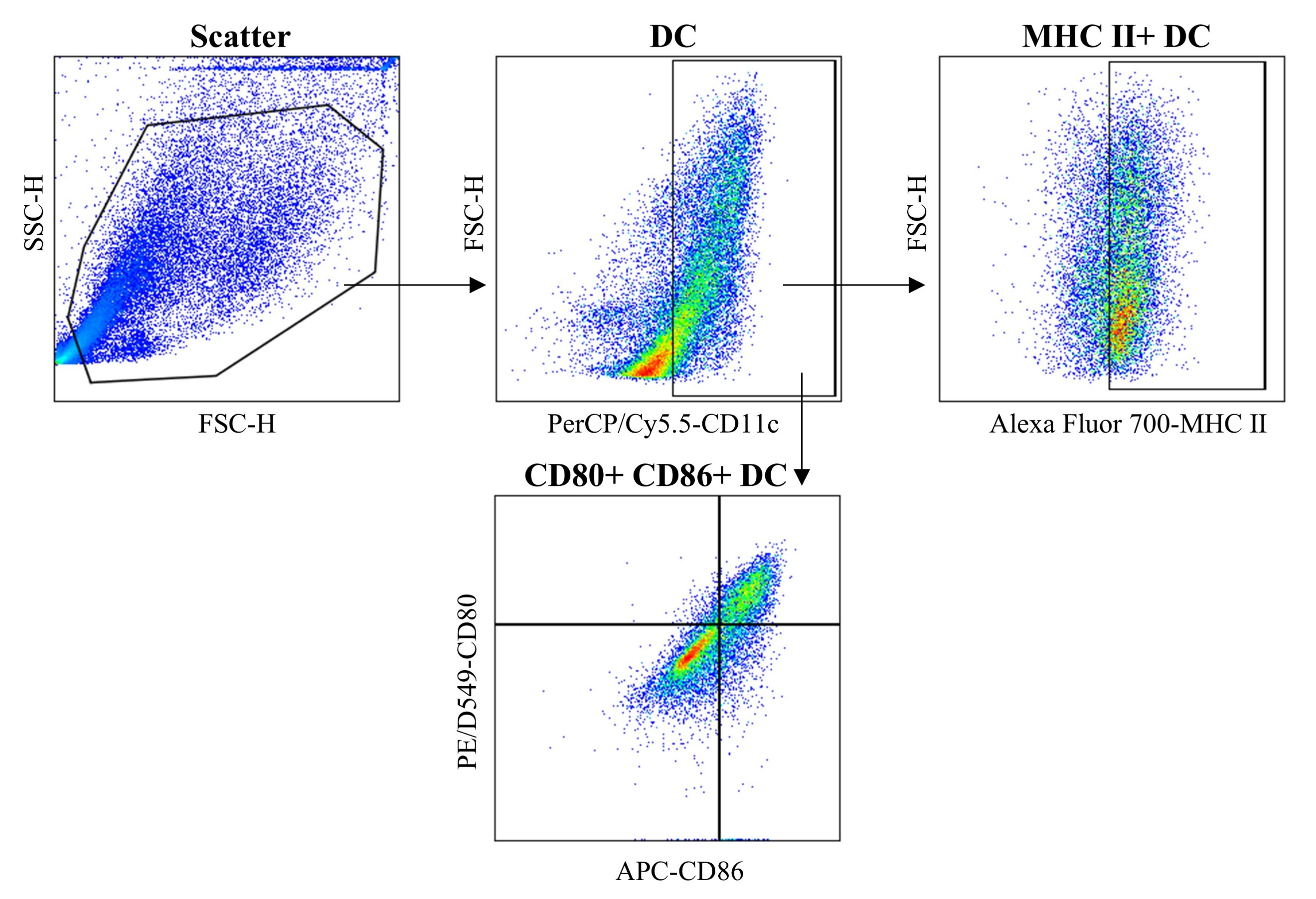


**Supplementary Figure 4.** Representative flow cytometry gating strategies for mature BMDCs (CD80+ CD86+) and MHC Ⅱ+ BMDCs.


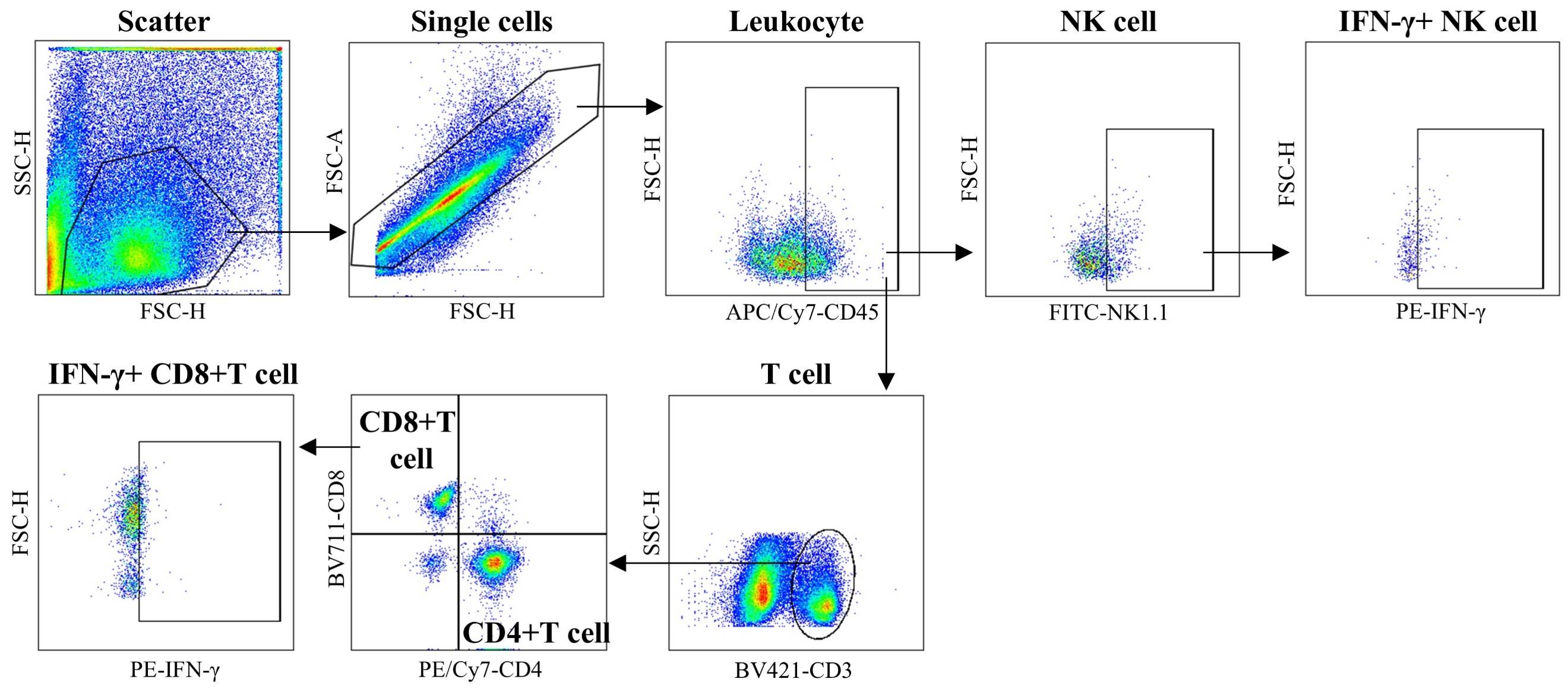


**Supplementary Figure 5.** Representative flow cytometry gating strategies for IFN-γ+ NK cells and IFN-γ+ CD8+ T cells in TME.
